# Attenuated Viral Replication of Avian *Infectious Bronchitis Virus* with a Novel 82-Nucleotide Deletion in the 5a Gene Indicates a Critical Role for 5a in Virus-Host Interactions

**DOI:** 10.1128/spectrum.01405-22

**Published:** 2022-06-29

**Authors:** Ye Zhao, Rong Liang, Jinlong Cheng, Jing Zhao, Jia Xue, Guozhong Zhang

**Affiliations:** a Key Laboratory of Animal Epidemiology of the Ministry of Agriculture, College of Veterinary Medicine, China Agricultural University, Beijing, China; University of Prince Edward Island

**Keywords:** γ-coronavirus, 5a gene, virulence, nuclear and cytoplasmic transport

## Abstract

We previously found that a deletion in γ-coronavirus *Infectious bronchitis virus* (IBV) accessory gene 5a is critical for decreased viral pathogenicity in chickens. Here, we systematically analyzed IBV virus infection: invasion, genome replication, subgenomic mRNA (sgmRNA) synthesis, protein synthesis, and virion release. The ability of the mutant IBV strain rYN-Δ5a to invade susceptible cells was not significantly different from that of parental rYN. However, compared with rYN, the level of sgmRNA synthesis and genome replication after cell entry by rYN-Δ5a was significantly lower in the early stage, resulting in a significantly lower level of nucleoprotein (N) synthesis and a consequent significantly lower number of offspring viruses released into the supernatant. The detected 5a protein was diffusely distributed in the cytoplasm and perinuclear area. We identified 16 differentially expressed host proteins, 8 of which were found to be host nuclear and cytoplasmic transport-related proteins. Coimmunoprecipitation revealed an interaction between hemagglutinin (HA)-tagged TNPO1, TNPO3, XPO1, XPOT, RanBP1, and EIF2B4 proteins and Flag-tagged 5a protein, and laser confocal microscopy confirmed 5a protein colocalization with these proteins, indicating that 5a protein can cause changes in the host protein localization. These host proteins promote the nuclear localization of N proteins, so we believe that 5a protein can hijack host nucleoplasmic transport-related proteins to help N enter the nucleus. This may involve regulating the cell cycle to promote the optimal intracellular conditions for virus assembly or by participating in the regulation of nucleolar function as a strategy to optimize virus replication.

**IMPORTANCE** Coronaviruses (CoVs) have a huge impact on humans and animals. It is important for the prevention and control of the viruses to assess the molecular mechanisms related to virulence attenuation. Here, we systematically analyzed a single cycle of virus infection by γ-CoV IBV lacking accessory protein 5a. We observed that a 5a deletion in the IBV genome affected virus replication and sgmRNA synthesis early in the virus life cycle, leading to decreases in protein synthesis, offspring virus assembly, and virion release in chicken embryonic kidney cells. IBV 5a protein was found to interact with multiple host nuclear and cytoplasmic transport- and translation-related proteins, which can also interact with IBV N and relocate it into the cell nucleus. These findings provide a comprehensive view regarding the importance of IBV accessory protein 5a and an important theoretical basis for studying the interaction between coronavirus and host cell proteins.

## INTRODUCTION

Coronaviruses are pathogens that seriously affect human and animal health (1). In recent years, zoonotic coronaviruses such as severe acute respiratory syndrome coronavirus (SARS-CoV), Middle East respiratory syndrome coronavirus (MERS-CoV), and SARS-CoV-2 (the causative pathogen for COVID-19) have had a serious impact on global economic and social development, and there may be more pathogenic coronavirus outbreaks in the future (2–4). However, at present, our options for the prevention and control or treatment of coronavirus infections are still very limited, highlighting the importance of improving our understanding of coronavirus virulence factors, replication characteristics, and host interactions. Given the challenges that SARS-CoV, MERS-CoV, and SARS-CoV-2 have posed, studies of coronavirus-specific proteins to enhance our understanding of coronavirus pathobiology are needed, and their findings may translate into new opportunities and targets for the design of antiviral therapeutics.

Avian infectious bronchitis (IB) is a highly infectious acute disease in chickens caused by avian *Infectious bronchitis virus* (IBV). This virus belongs to the order *Nidovirales*, family *Coronavirus*, coronavirus group III (5). IBV is a positive-stranded RNA (+RNA) virus with a large genome of 27 kb. It has a polycistronic genome organization and employs a unique transcription mechanism to generate a nested set of subgenomic mRNAs (sgmRNAs). These are used to express the open reading frames (ORFs) located downstream of the replicase ORFs 1a and 1b, which encode structural and accessory proteins. The sgmRNAs are 3′ coterminal, but they also contain a common 5′ leader sequence. The leader and “body” segments of the sgRNA are joined during discontinuous negative-strand RNA synthesis, which produces a subgenome-length template for each of the sgmRNAs (6). The IBV genome harbors two group-specific genes, genes 3 and 5, which are functionally tricistronic and bicistronic, respectively, and can be translated into four accessory proteins (3a, 3b, 5a, and 5b) and structural protein E with a verified transcription regulatory sequence (TRS) identified upstream of ORFs 3 and 5 (7). These accessory proteins offer functional flexibility to coronaviruses and are subject to alterations depending upon the condition in which they are expressed during the viral life cycle.

Since IBV was first discovered in the 1930s, it has spread widely around the world, causing serious harm and inestimable economic losses to the poultry industry (8). In the late 1990s, the epidemic situation of IBV changed greatly. The occurrence of large-scale outbreaks of IB in chickens immunized against IBV indicates that the antigenicity of IBV in China may have mutated. The results of immune challenge tests, serum neutralization tests, and epidemiological investigations of specific-pathogen-free (SPF) chickens all proved that H120 and other commercially available vaccines could provide only partial protection, if any. To address this situation, the QX type IBV strain YN was isolated from an immunized chicken flock and continuously passaged on in SPF chicken embryos for 160 generations in our lab. The results of an infection test of 5-week-old SPF chickens showed that there was a significant difference in pathogenicity between the parent strain YN and the attenuated strain aYN, even though the sequence homology between these strains was as high as 99.7%. This finding suggested that something in the 0.3% of gene loci that were differentially expressed was the key to the altered virulence of the attenuated virus (9).

By comparing the sequence differences, an interesting feature was observed in the genome of aYN; it was found that there was a continuous natural deletion of 82 nucleotides (nt) toward the 5′ region of ORF5, leading to a failure of ORF translation for the 5a gene. By applying reverse genetics, we used the YN strain as a platform to modify the 82-nt deletion region of the 5a gene, successfully constructing and saving the IBV deletion mutant virus rYN-Δ5a. The virulence of rYN-Δ5a in SPF chickens was significantly lower than that of the parental YN strain, suggesting that the deletion of 5a may affect IBV virulence (10). The purpose of the present study is to investigate the precise role of a 5a deletion on the virulence of IBV by assessing two aspects: comparison of virus replication over a single life cycle and host interaction with 5a protein. We anticipate the findings will provide direction for the development of new vaccine strains via the rapid and accurate transformation of coronaviruses. The results will also serve as important reference values for future studies of the IBV pathogenic mechanism and the development of attenuated virus for use as a vaccine.

## RESULTS

### The 5a gene is critical for the efficient replication of IBV in chicken embryonic kidney (CEK) cells.

**FIG 1:**
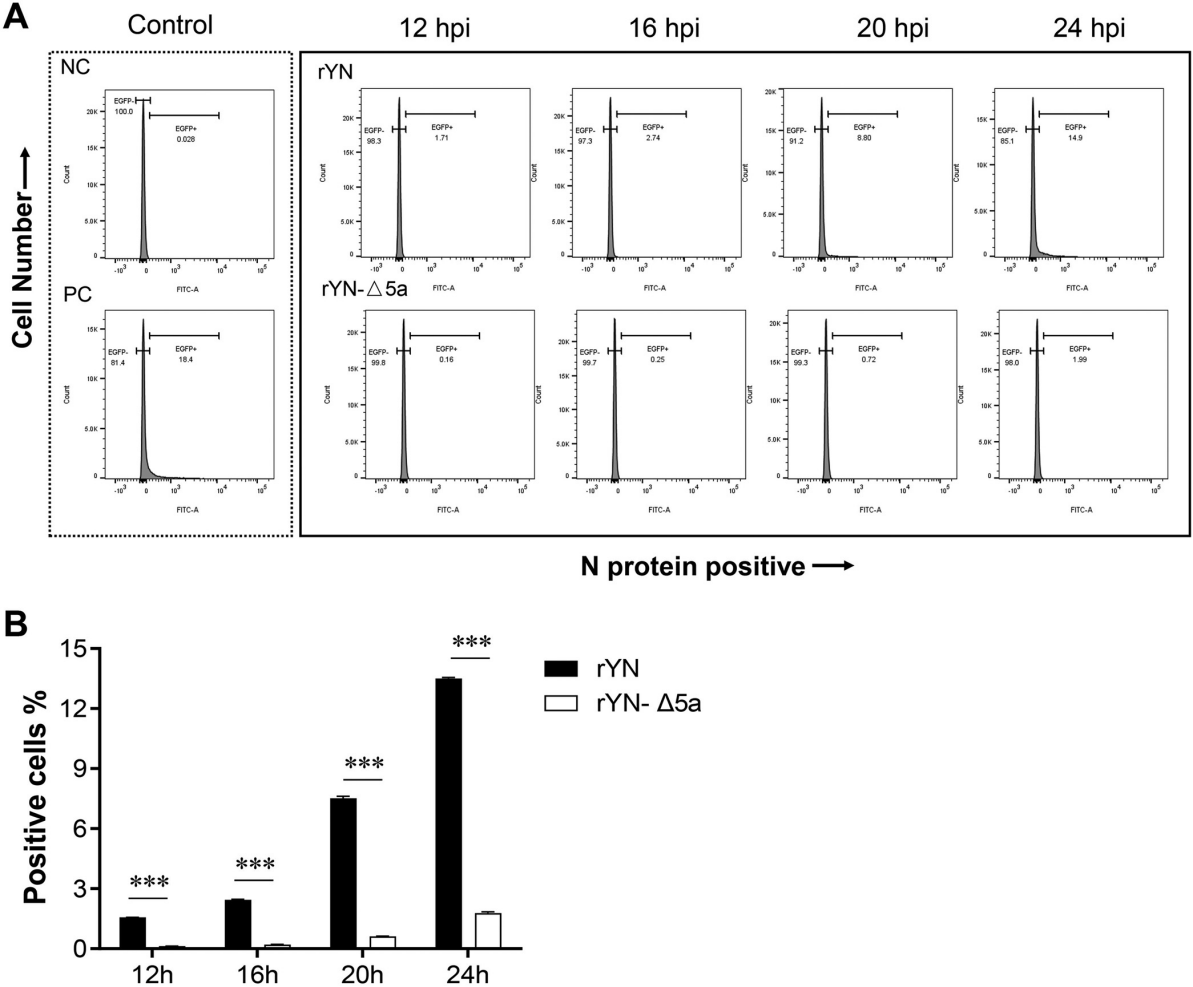
Flow cytometry analysis of IBV N-positive cells. CEK cells were infected with rYN or rYN-Δ5a, then stained at various time points with mouse antibodies specific for the IBV structural protein N, and subjected to flow cytometry to measure the percentage of EGFP-positive (infected) cells. The background fluorescence of the cells was subtracted based on data from uninfected cells. Artificially constructed IBV-EGFP viruses were used as a positive control. At each time point, three samples from each group were tested, and the mean infected cell percentages were statistically evaluated using a two-way ANOVA test adjusted for *post hoc* analysis, followed by Bonferroni’s multiple-comparison tests. Statistically significant differences between groups are highlighted; *, *P* < 0.05; **, *P *< 0.01; ***, *P* < 0.001.

To test whether an 82-nt deletion in IBV 5a restricts viral entry and early-stage replication, we compared the percentages of CEK cells infected by rYN or rYN-Δ5a at different time points (2, 4, 6, 8, 12, 16, 20, or 24 h postinfection [hpi]) using flow cytometry (Fig. 1A). In the first 8 h after infection, only a very small number of cells were infected with either virus, and there were no significant differences in the percentage of infected cells between the rYN and rYN-Δ5a groups (data not shown), which indicates that there is no difference in the viral entry stages of these two viruses. At 12 hpi, obvious differences were detected in the viral replication of rYN and rYN-Δ5a; in the rYN-infected group, 1.71% of CEK cells were IBV-positive, whereas only 0.15% of CEK cells were IBV-positive in the rYN-Δ5a-infected group. At 16, 20, and 24 hpi, the percentage of IBV-positive cells in the rYN group gradually increased to 2.74%, 8.80%, and 14.9%, respectively, all of which were significantly higher than the corresponding IBV-positive percentages in the rYN-Δ5a-infected group (0.25%, 0.72%, and 1.99%, respectively) (Fig. 1B).

**FIG 2:**
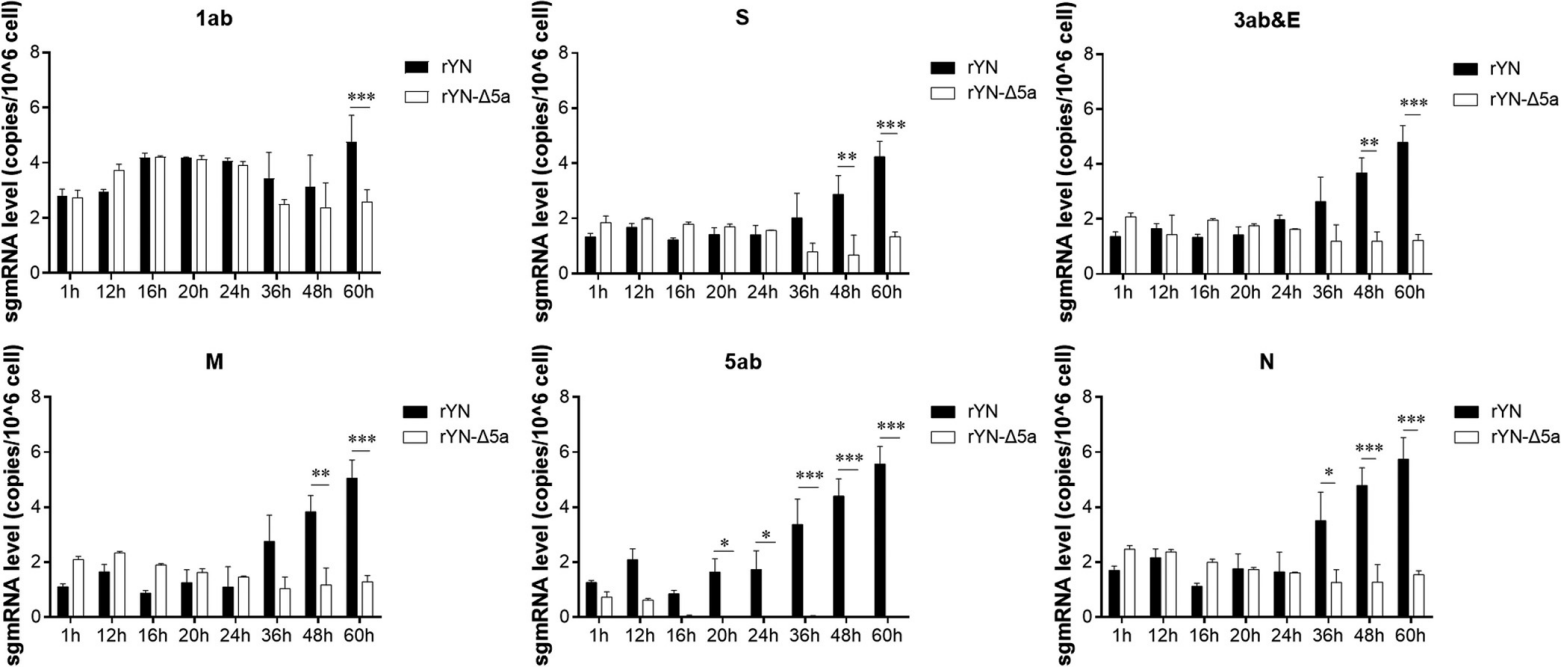
Genomic RNA and sgmRNA copy numbers in CEK cells infected with rYN or rYN-Δ5a. CEK cells were inoculated with rYN or rYN-Δ5a and then harvested at various time points for use in qRT-PCR to measure the genomic RNA and sgmRNA copy numbers. Leader-F was used as the forward primer, and different reverse primers were used to measure the genome replication (1ab) and sgmRNA synthesis (S, 3ab&E, M, 5ab, and N) levels (see Table 2). All assays were run in triplicate, and sample copy numbers were calculated based on a standard curve. Data were statistically evaluated by a two-way ANOVA test adjusted for *post hoc* analysis, followed by Bonferroni’s multiple-comparison tests. Statistically significant differences between groups are highlighted; *, *P* < 0.05; **, *P* < 0.01; ***, *P < *0.001.

To determine the genome replication efficiency and sgmRNA synthesis level of rYN and rYN-Δ5a, specific primers for 1ab and sgmRNA of various lengths were designed and used in real-time quantitative (qRT)-PCR to analyze the total RNA extracted from CEK cells that had been infected with rYN or rYN-Δ5a for 1, 12, 16, 20, 24, 36, 48, or 60 h. By 24 hpi, except for the expected negative 5a-sgmRNA quantitative PCR (qPCR) detection in the rYN-Δ5a infection group caused by the 5a gene deletion, there was no significant difference in the copy numbers of the virus genome or other subgenomes between the two infection groups. At 36 hpi, the genome replication level and sgmRNA synthesis level of the rYN-Δ5a infection group were obviously lower than those of the rYN infection group, among which the difference in sgmRNA-N synthesis level was the largest. At 48 hpi, the sgmRNA synthesis level in the rYN-Δ5a infection group was significantly lower than that in the rYN infection group, and there was also a significant difference in the genome replication level at 60 hpi (Fig. 2).

**FIG 3:**
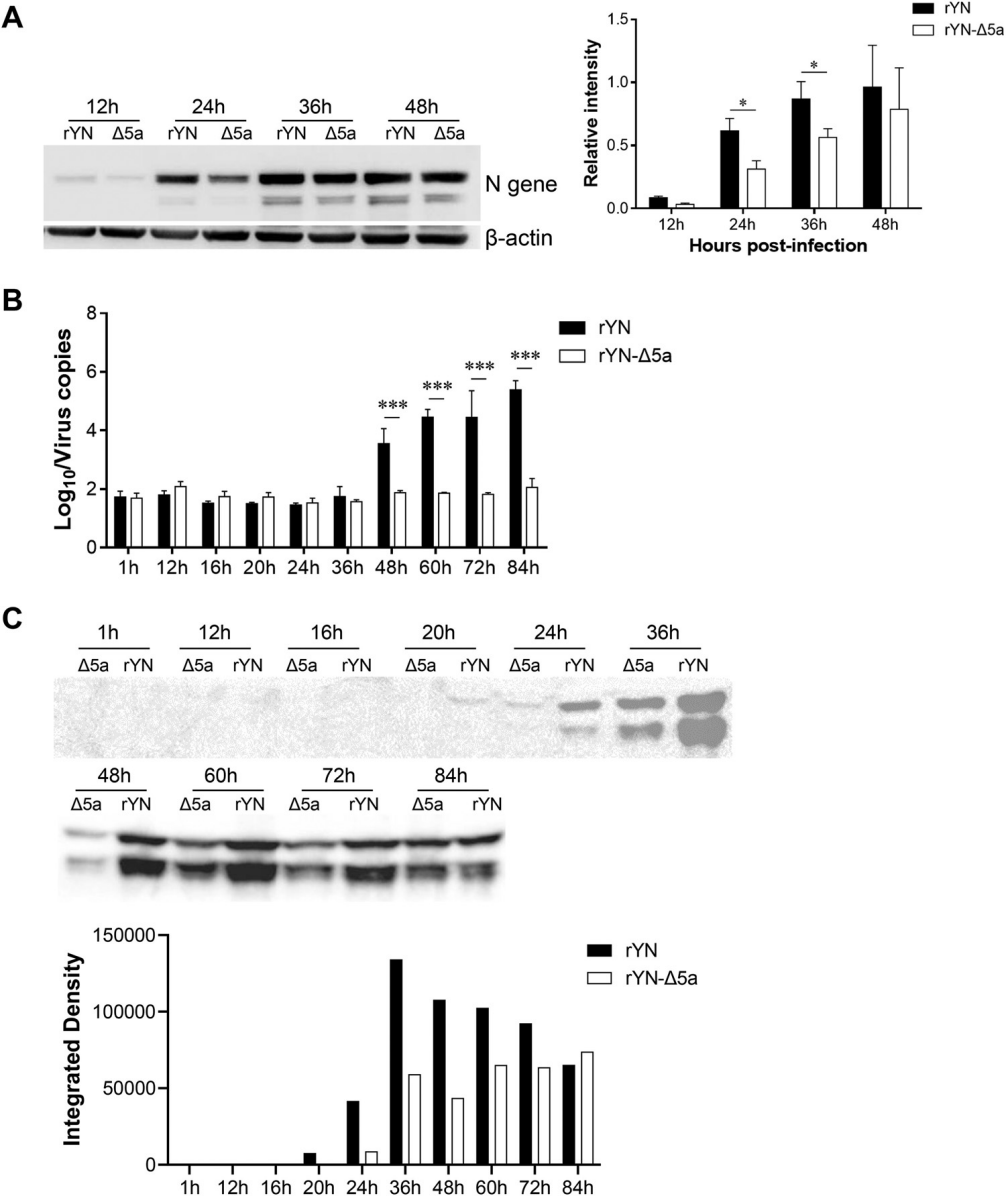
N synthesis and offspring virus release into the supernatant in CEK cells infected with rYN or rYN-Δ5a. CEK cells were inoculated with rYN or rYN-Δ5a. (A) Representative Western blot analysis of whole-cell lysates using antibodies recognizing IBV N and β-actin. The expression level of IBV N was quantified by densitometry and normalized to the β-actin expression level. Results shown are the means of three independent determinations (± standard error of the mean [SEM]). *, *P* < 0.05. (B) qRT-PCR with primers Leader-F and 1ab-R was conducted on the supernatant of cells harvested at various time points postinfection. All assays were run in triplicate, and values were calculated based on a standard curve. Data were statistically evaluated by a two-way ANOVA test adjusted for *post hoc* analysis, followed by Bonferroni’s multiple-comparison tests. Statistically significant differences between groups are highlighted; *, *P* < 0.05; ***, *P* < 0.001. (C) Representative Western blot analysis of the supernatant proteins at various time points using antibodies recognizing IBV N. The N expression levels were taken as a proxy measurement of released offspring virus. The expression level of IBV N was quantified by integrated density.

To compare the protein expression levels of rYN and rYN-Δ5a, the protein synthesis levels of N for rYN and rYN-Δ5a at different time points after infection of CEK cells were determined by Western blotting. β-actin was used as an internal control, and the results of three repeated experiments were quantified and analyzed. The protein expression level of N in the rYN-Δ5a infection group was lower than that in the rYN infection group for each time point after infection, with the differences at 24 hpi and 36 hpi being the largest (Fig. 3A).

To compare the numbers of offspring viruses released into the supernatant between rYN and rYN-Δ5a, we estimated the viral amount based on the levels of RNA and protein. Before 36 hpi, only a small amount of viral RNA was detected in the supernatant by qRT-PCR for either virus, and no difference was detected between rYN and rYN-Δ5a. At 48 hpi, a large amount of viral RNA was detected in the supernatant of the rYN infection group, whereas the amount of viral RNA in the supernatant of the rYN-Δ5a infection group was almost undetectable (Fig. 3B). At the protein level, the offspring viruses could be detected as early as 20 hpi for rYN and 24 hpi for rYN-Δ5a. The level of N released by rYN-Δ5a offspring viruses was lower than that of rYN offspring viruses at each time point through at least 84 hpi (Fig. 3C).

### IBV 5a gene interacts with various host nuclear and cytoplasmic transport proteins.

**FIG 4:**
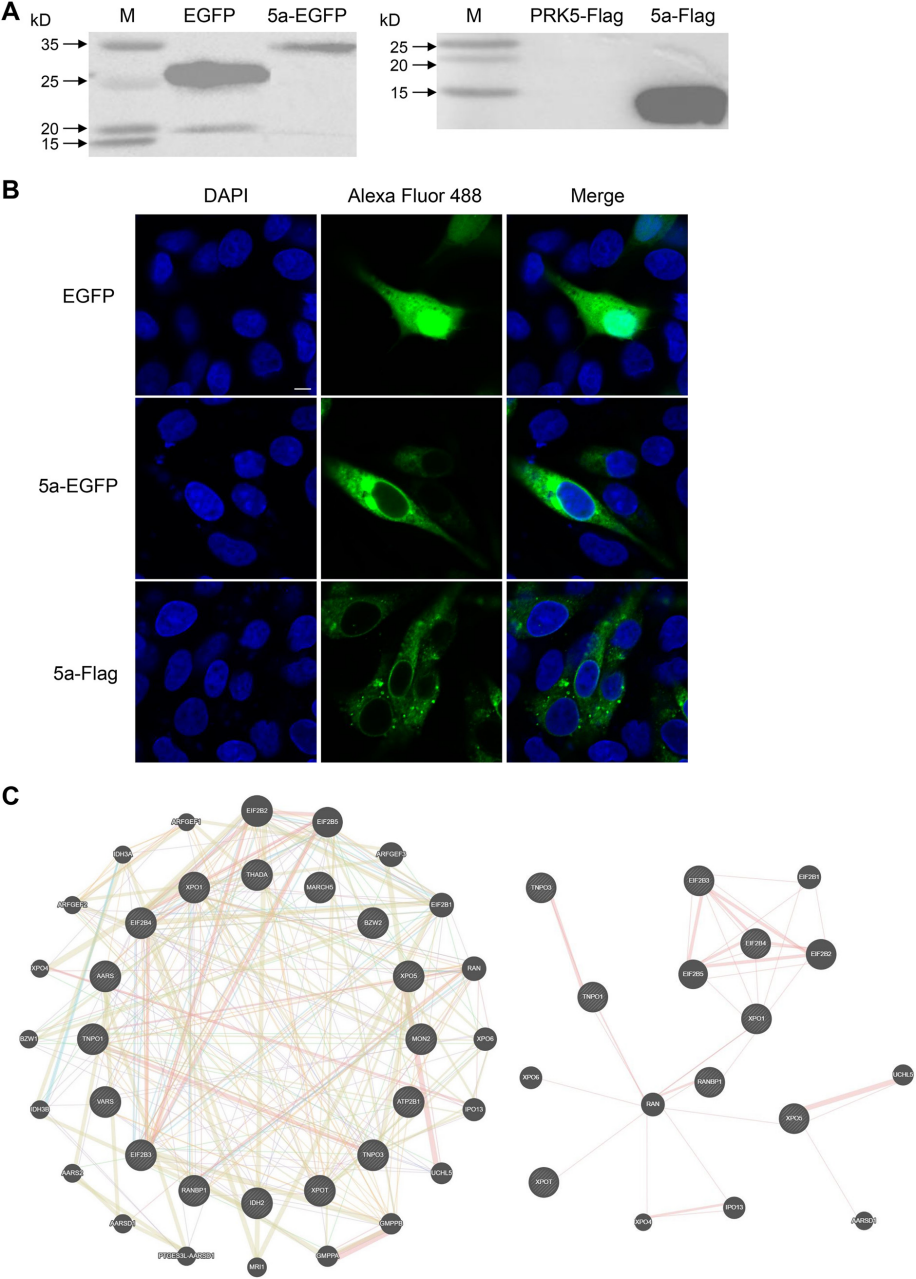
Expression, cellular sublocalization pattern, and interacting host proteins of EGFP-tagged and Flag-tagged 5a fusion protein. Vero cells were transfected with eukaryotic expression vectors for EGFP- or Flag-tagged 5a protein. (A) Western blot analysis of whole-cell lysates using antibodies recognizing EGFP or Flag-tag. (B) The sublocalization pattern of 5a protein in the cells was observed under a confocal microscope through EGFP fluorescence or ani-Flag antibodies in Vero cells transfected with pRK5-5a-EGFP or pRK5-5a-Flag, respectively. The results were observed at 24 h posttransfection. Nuclei were counterstained with DAPI (blue). Scale bar = 20 μm. (C) The constructed protein-protein interaction (PPI) network for 5a protein-correlated host proteins. The functional annotation of the genes included in the right-side nodules are listed separately in Table 2.

The significant differences observed between the replication efficiency of rYN and rYN-Δ5a indicate that the IBV 5a gene plays an important role in the virus life cycle. To investigate the specific function of 5a in the host cells, we constructed eukaryotically expressed 5a protein fused with an enhanced green fluorescent protein (EGFP) tag or Flag tag by using PCR cloning and homologous recombination. The recombinant plasmids were transfected into Vero cells after the correct sequences were verified by sequencing, and the resulting total cell protein was harvested. The size of the EGFP-tagged 5a protein was about 36 kDa, while that of EGFP protein alone is 26 kDa, and the size of the Flag-tagged 5a protein was about 11 kDa, while the Flag tag was not detected in the control group transfected with pRK5-Flag vector (Fig. 4A). The results show that both EGFP-tagged and Flag-tagged exogenous 5a protein can be expressed normally in Vero cells.

Vero cells were transfected with the eukaryotic expression vectors for EGFP- or Flag-tagged 5a protein. The results show that the tagged 5a proteins are expressed at the predicted correct molecular weights (Fig. 4A). The sublocalization pattern of 5a protein in the cells was observed. Confocal microscopy revealed that the 5a protein was diffusely distributed in the cytoplasm and perinuclear area but excluded from the nucleus, whereas the control EGFP protein was diffusely distributed in both the nucleus and cytoplasm without specific localization. In addition, we observed that there appeared to be minor localization differences between the different tagged 5a proteins; 5a-EGFP is possibly backed up in the endoplasmic reticulum (ER), whereas 5a-Flag is cytoplasmic and punctate, which we believe is mainly due to permeabilization during detection of the Flag tag, whereas the EGFP-5a was observed directly under a fluorescence microscope (Fig. 4B).

To identify the host proteins that interact with exogenous 5a protein, PRK5-Flag-5a was transfected into Vero cells, and the total protein was extracted for use in a coimmunoprecipitation (co-IP) assay. The pRK5-Flag transfection group was set as a negative control. The resulting SDS-PAGE gel was stained with Coomassie brilliant blue. Differences between the experimental group and the control group mainly ranged from 25 to 60 kDa and 100 to 180 kDa. Different gel strips were sent away for mass spectrometry identification. Based on an analysis of the number of peptide segments, 16 cellular proteins were identified as interacting with 5a protein (Fig. 4C, left). Through Go Gene Ontology (GO) annotation and GeneMANIA analysis, it was found that eight of these host proteins are closely related to host nucleocytoplasmic transport (Fig. 4C, right). Specific information regarding the above proteins is shown in Table 1.

**TABLE 1:** Details of the nuclear and cytoplasmic transport-related host proteins found to interact with IBV 5a

UniProt Protein ID	Protein	Gene	Size (kDa)	GO annotation
A0A0D9RQ38	Exportin 1	XPO1	123	Protein localization to nucleus; regulation of centrosome duplication, protein catabolic process, and regulation of protein export from nucleus; ribosomal subunit export from nucleus; nuclear export signal receptor activity; Ran GTPase binding
A0A0D9QWP5	Exportin-T	XPOT	55	tRNA reexport from nucleus; Ran GTPase binding; tRNA binding
A0A0D9RHM3	Exportin 5	XPO5	136	Positive regulation of RNA interference; pre-miRNA export from nucleus; protein export from nucleus; mRNA binding; nuclear export signal receptor activity; pre-miRNA binding; Ran GTPase binding; RNA-induced silencing complex (RISC) complex binding; tRNA binding
A0A0D9RSW2	Transportin 1	TNPO1	102	Protein import into nucleus; Ran GTPase binding
A0A0D9RC70	Transportin 3	TNPO3	104	Protein import into nucleus; identical protein binding; Ran GTPase binding
A0A0D9S7G7	Eukaryotic translation initiation factor 2B subunit gamma	EIF2B3	46	Oligodendrocyte development; T cell receptor signaling pathway; guanyl-nucleotide exchange factor activity; translation initiation factor activity; eukaryotic translation initiation factor 2B complex
A0A0D9RBT6	Eukaryotic translation initiation factor 2B subunit delta	EIF2B4	57	Cellular metabolic process
A0A0D9RHX8	RanBD1 domain-containing protein	RanBP1	30	Intracellular transport; positive regulation of mitotic centrosome separation; spindle organization

### 5a protein affects the cellular localization of host nucleocytoplasmic transport proteins.

**FIG 5:**
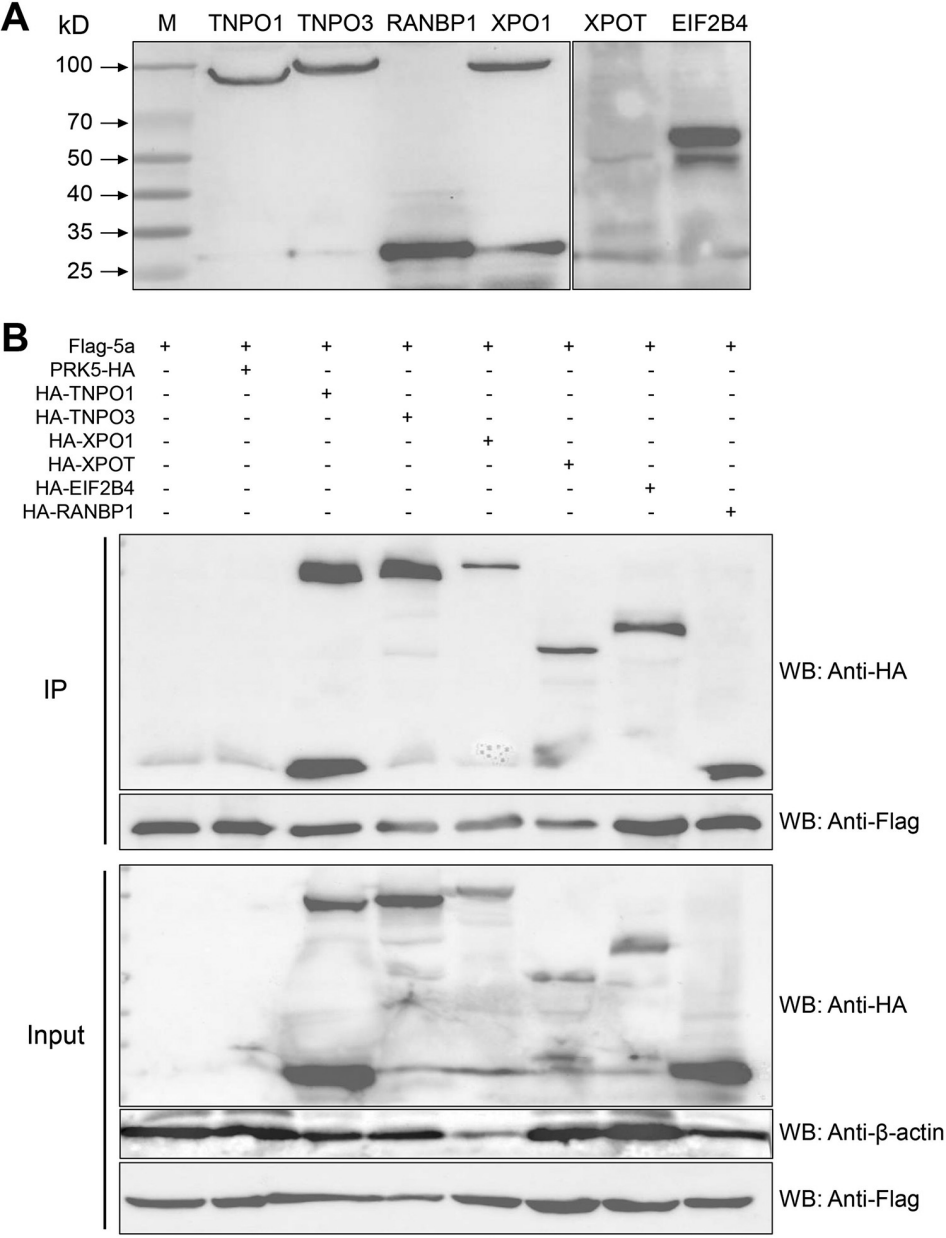
Expression and co-IP of HA-tagged XPOT, XPO1, TNPO1, TNPO3, RanBP1, and EIF2B4 with Flag-tagged 5a fusion protein. (A) Western blot analysis of whole-cell lysates of 293T cells transfected with eukaryotic expression vectors for HA-tagged XPOT, XPO1, TNPO1, TNPO3, RanBP1, or EIF2B4, probed with antibodies recognizing HA-tag. (B) Co-IP blots of anti-Flag antibody immunoprecipitations from 293T cells cotransfected with eukaryotic expression vectors for HA-tagged XPOT, XPO1, TNPO1, TNPO3, RanBP1, or EIF2B4 proteins and for Flag-tagged 5a, probed with anti-Flag and anti-HA antibodies. An unlabeled, antibody-coupled protein A/G-coupled agarose affinity gel was used as the negative control for nonspecific binding.

Based on the GO annotation results for the identified host proteins, six host proteins (TNPO1, TNPO3, RanBP1, XPO1, XPOT, and EIF2B4) were chosen for reverse co-IP reconfirmation and colocalization analysis. 293T cells were transfected with eukaryotic expression vectors for hemagglutinin (HA)-tagged TNPO1, TNPO3, RanBP1, XPO1, XPOT, or EIF2B4. After transfection, the total cell proteins were harvested, and their expression and size were verified by Western blotting. The HA-tagged TNPO1, TNPO3, RanBP1, XPO1, XPOT, and EIF2B4 proteins were expressed normally in 293T cells, and their sizes are consistent with the expected protein size, indicating the successful construction of eukaryotic expression plasmids for these six HA-tagged host proteins, which can be used in further experiments (Fig. 5A).

**FIG 6:**
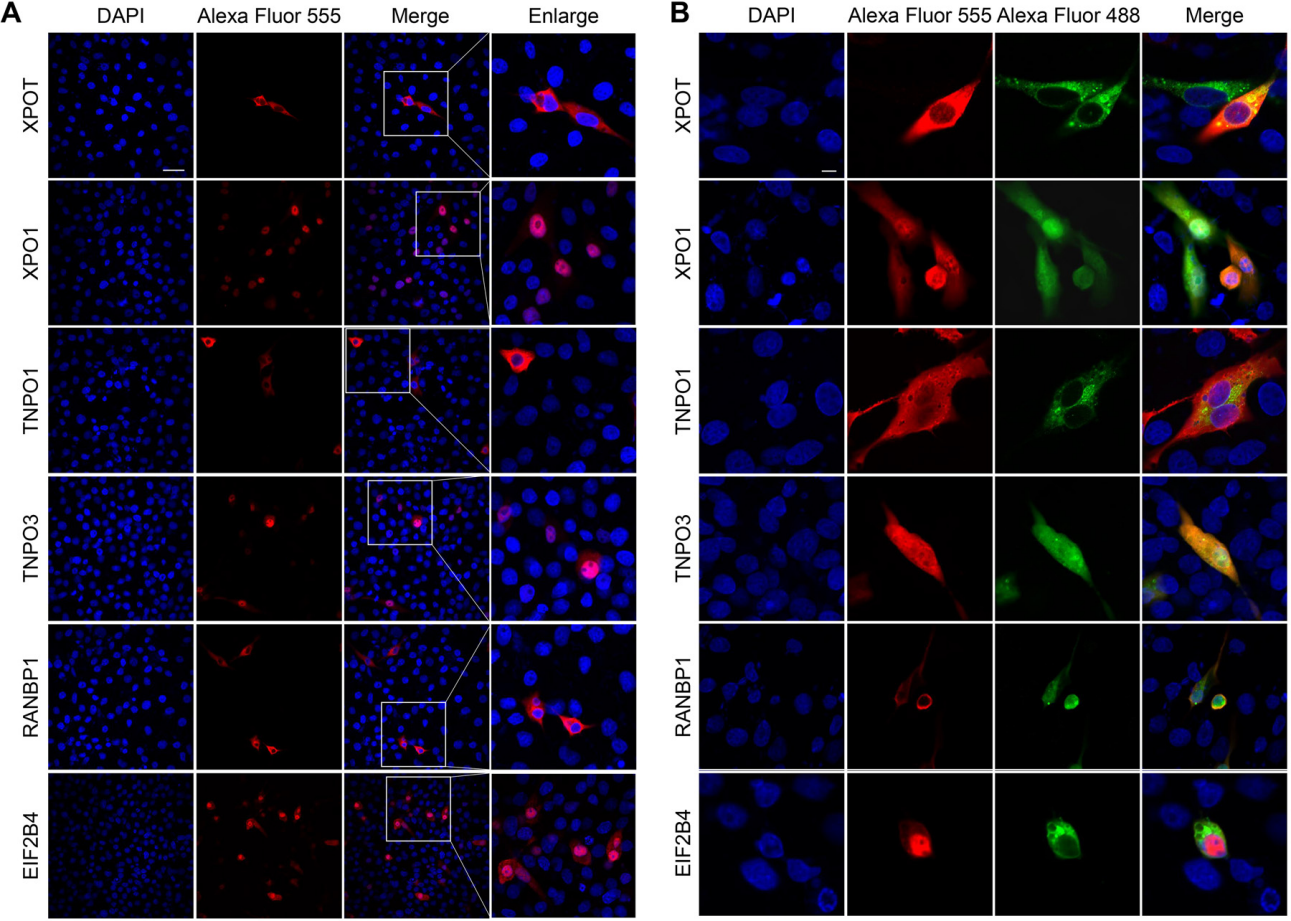
Cellular sublocalization pattern of HA-tagged XPOT, XPO1, TNPO1, TNPO3, RanBP1, and EIF2B4 in the presence or absence of 5a protein. (A and B) Vero cells were transfected with eukaryotic expression vectors for HA-tagged XPOT, XPO1, TNPO1, TNPO3, RanBP1, or EIF2B4 proteins alone (A) or were cotransfected with those expression vectors and an expression vector for Flag-tagged 5a (B). At 48 h posttransfection, paraformaldehyde-fixed, cyro-embedded cells were stained with antibodies against Flag (green) or HA (red) tag. Nuclei were counterstained with DAPI (blue). Images were captured using a Nikon A1 confocal microscope. Scale bar = 10 and 20 μm in panels A and B, respectively.

The constructed HA-tagged host protein expression plasmids were cotransfected with pRK5-Flag-5a into 293T cells. The host protein was detected by co-IP and Western blotting at 36 h posttransfection. Unlabeled antibody-coupled protein A/G-coupled agarose affinity gel was used as the negative control for nonspecific binding. The coprecipitation of all host proteins with 5a protein could be detected by Flag-labeled antibody coupled with agarose agglutination; the expression of specific host protein and 5a protein could not be detected in the negative-control group (Fig. 5B). pRK5-HA-TNPO1, -TNPO3, -RanBP1, -XPO1, -XPOT, and -EIF2B4 were each separately transfected into Vero cells, and the localization of these host proteins in the cells was detected by laser confocal microscopy. XPOT, TNPO1, and RanBP1 were distributed in the cytoplasm, TNPO3 and EIF2B4 were predominantly distributed in the nucleus, with some expression in the cytoplasm, and XPO1 was limited to the nucleoplasm (Fig. 6A).

Confocal microscopy was used to detect the colocalization of 5a protein and host protein in Vero cells at 48 h after cotransfection. 5a protein was colocalized with each of the six host proteins, and a small amount of XPOT and TNPO1 were transferred from the cytoplasm to the nucleus. Although TNPO3 were still distributed in both the cytoplasm and the nucleus, their nuclear localization preference disappeared in the presence of 5a protein, and there was no difference in the amount of protein between the cytoplasm and the nucleus. The distribution patterns of XPO1 and RanBP1 also changed; the nucleoplasm-only localization pattern of XPO1 disappeared, while RanBP1 showed the phenomenon of cell membrane aggregation. Only the localization of EIF2B4 did not change, and most of them were still located in the nucleoplasm; only a few were in the cytoplasm (Fig. 6B).

### 5a protein affects the cellular localization of IBV N in the state of infection or transfection.

We identified a variety of host proteins that interact with 5a protein, several of which are related to nuclear and cytoplasmic transport. Thus, we further studied whether the IBV N that can localize in the host nucleus is affected by 5a protein via its interaction with these nuclear and cytoplasmic transport proteins. The cellular location of IBV N protein with/without the existence of 5a was examined under infection and transfection, respectively. Upon infection, CEK cells were infected by rYN or rYN-Δ5a; the localization of N protein was qualitatively and quantitatively analyzed by immunofluorescence and nuclear-cytoplasmic separation assay, respectively. At the same time, we inversely verified the key role of 5a protein in N protein localization by electroporation of Flag-tagged 5a plasmid to supplement 5a protein and set up Flag-tag plasmid as a negative control. Upon transfection, Vero cells were transfected with IBV-N expression plasmid and cotransfected with Flag-tagged 5a protein or host protein expression plasmids, and we observed whether there were similar localization changes visible by confocal microscopy.

**FIG 7:**
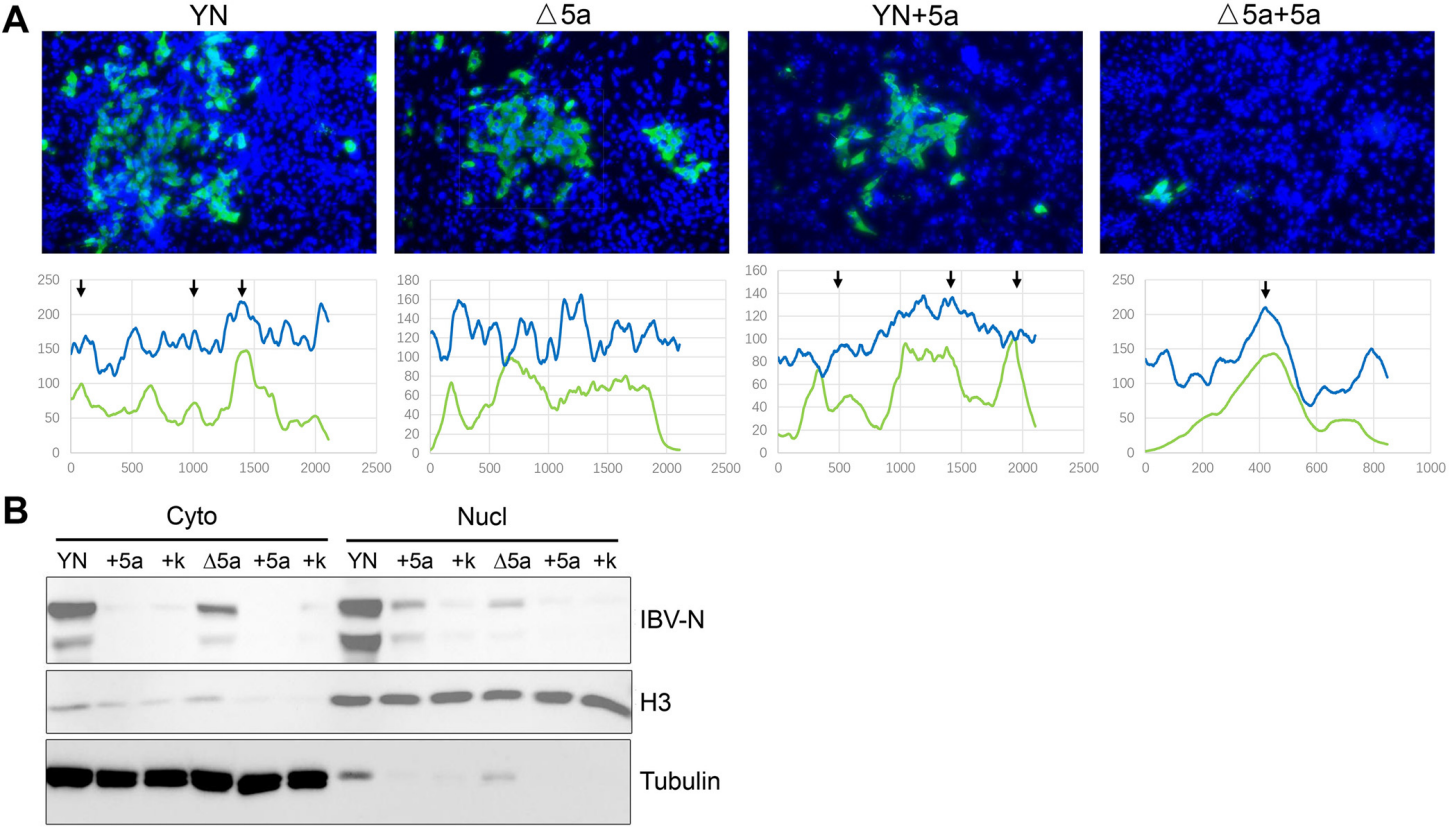
Cellular sublocalization pattern of IBV N in rYN or rYN-△5a infected CEK cells. CEK cells were inoculated with rYN or rYN-Δ5a. Expression plasmid for Flag-tagged 5a protein was electroporated into CEK cells after infection to supplement additional 5a protein (+5a). Flag-tag plasmid was set as the negative control (+k). (A) Sublocalization of IBV-N protein. Infected CEK cells were stained for IBV-N with anti IBV-N antibody (green) at 24 hpi. Nuclei were stained with DAPI (blue). Cells were analyzed by Nikon fluorescence microscopy using a 20× objective, and representative images are shown. Below the image is a profile showing the intensity of EGFP (green line) and DAPI (blue line) fluorescence in the image. (B) Expression of IBV-N protein in the cytoplasmic and nuclear extracts of CEK cells. Cytoplasmic and nuclear extracts of infected CEK cell lysates were prepared using a nuclear and cytoplasmic protein extraction kit and analyzed by Western blotting with anti-IBV-N antibody. Cytoplasmic extraction was analyzed using α-tubulin antibody, and nuclear extraction was analyzed using histone H3 antibody.

Under the fluorescence microscope, we observed that the change of 5a protein on the localization of N protein was consistent regardless of the state of infection or transfection. In CEK cells infected by rYN strain, we observed that there were two localization patterns of N protein, one part of which was in the cytoplasm, and another part was colocated with the nucleus (Fig. 7A). The results of nuclear and cytoplasmic separation showed that the amount of N protein distributed in the nucleus was similar to that in the cytoplasm (Fig. 7B). In the absence of 5a protein, most of the N proteins were distributed in the cytoplasm, and the phenomenon of colocalization with the nucleus disappeared. The results of Western blot analysis also showed that the N protein in the nucleus decreased significantly in the CEK cells infected with rYN-Δ5a strain. After the supplementation of 5a protein, the N protein in the nucleus increased, but due to the greater damage after transfection of primary CEK cells, the overall infection rate of IBV decreased significantly, so the expression of N protein decreased significantly.

**FIG 8:**
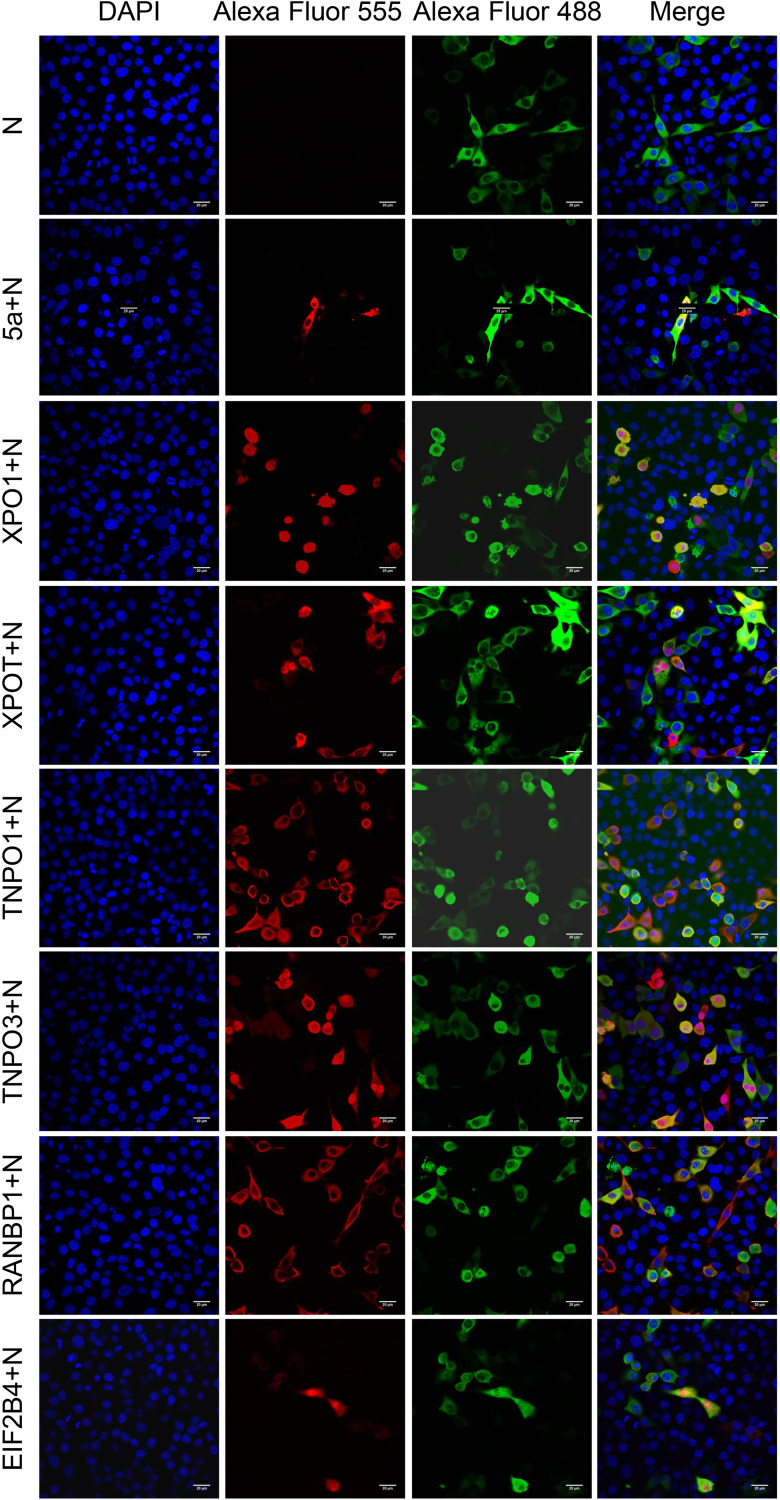
Cellular sublocalization pattern of IBV N in the presence or absence of 5a, XPOT, XPO1, TNPO1, TNPO3, RanBP1, and EIF2B4. Vero cells were transfected with PCI-N alone or were cotransfected with PCI-N and expression vectors for Flag-tagged 5a or HA-tagged XPOT, XPO1, TNPO1, TNPO3, RanBP1, or EIF2B4. At 48 h posttransfection, paraformaldehyde-fixed, cyro-embedded cells were stained with antibodies against Flag tag (red) and IBV N (green) or HA tag (red). Nuclei were counterstained with DAPI (blue). Images were captured using a Nikon A1 confocal microscope. Scale bar = 20 μm.

Following the transfection of IBV N alone, N was distributed predominantly in the cytoplasm, and the cells showed a normal spindle shape. After its cotransfection with 5a protein, the location of N changed; it accumulated in the nucleus, and the cell morphology also changed, with the cells appearing shrunken. These changes in cell localization pattern and cell morphology were also observed after the cotransfection of N with host nucleocytoplasmic transport-related proteins; both the localization of N and the cell morphology changed after cotransfection with XPO1, TNPO1, TNPO3, or RANBP1, while there was no change in cell morphology after cotransfection with XPOT or ELF2B4 (Fig. 8).

## DISCUSSION

Although the known pathological proteins spike and nucleoprotein are the targets of current therapeutic approaches for the control of coronavirus infection, it is imperative to explore other viral factors. Accessory proteins play group-specific roles in coronavirus pathobiology, and the genes for these proteins differ in genomic location, number, and nature between coronavirus groups, leading to their description as “group-specific genes” (11, 12). Although considered to be dispensable for viral replication and growth, the presence and maintenance of these genes within coronavirus genomes has led to huge interest in understanding their significance for viral life cycle and virulence (13). Presently, the specific mechanisms by which 5a protein functions in viral virulence, replication, and modification of the host antiviral response are still unclear. Here, to contribute to a holistic understanding of IBV accessory protein ORF5a, we analyzed the replication characteristics and host interactions of this protein.

After entering a host cell, IBV goes through many life cycle stages, such as genome replication, sgmRNA synthesis, protein synthesis, virus assembly, and virus release (14). To understand the specific role of 5a protein in IBV replication, it is important to determine which stage(s) of the virus replication cycle is/are affected by the deletion of 5a protein. Our flow cytometry results showed that the percentage of infected cells was significantly higher than that in the rYN-Δ5a infection group, indicating that the early replication ability of the virus was significantly inhibited after cell entry.

The sgmRNA synthesis level of the rYN-Δ5a infection group was lower than that of the rYN group, with the largest difference seen in the sgmRNA N synthesis levels. Correspondingly, the protein expression level of IBV N in the rYN-Δ5a infection group was lower than that in the rYN group at each time point after infection, with the largest difference observed at 24 and 36 hpi. These findings indicate that the level of sgmRNA synthesis by rYN-Δ5a was significantly inhibited after cell entry, resulting in a significantly lower level of protein synthesis than that of rYN.

These differences in the number of released offspring viruses were mirrored by similar differences in the protein expression level of N, although N expression was detected as early as 20 hpi in the rYN infection group. The timing differences between the RNA and protein expression levels may result from the sensitivity diversity of the detection methods. There was a significant difference in the genome replication level at 60 hpi, which was probably due to a significant decrease in the number of offspring viruses in the cell and the number of offspring viruses released into the supernatant.

In previous studies, researchers found a truncation of ORF8 (29-nt deletion) in SARS-CoV, which affected early-stage transmission and decreased viral replication up to 23-fold (15, 16). The genetic and functional importance of this 29-nt deletion in SARS-CoV ORF8ab has been a hotly debated issue. Whether it is the result of genomic instability or adaptive evolution, its presence in the SARS-CoV genome has been assumed to contribute to the zoonosis transformation and human adaptation of the virus. This contribution to human adaptation has been attributed to the corresponding missing function, which may lead to the development of proteins with new functions in the later stages of a SARS-CoV epidemic. Alternatively, based on the founder effect, the deletion may allow SARS-CoV to survive with reduced adaptability and might not play a universal role in SARS-CoV host adaptation (15). Genome deletions have also been observed in MERS-CoV; these were speculated to reflect adaptation to the human host or the release of a selective pressure exerted exclusively in the zoonotic reservoir (17, 18). The 82-nt deletion in IBV studied here was found following continuous passaging of IBV in chicken embryos, and this deletion attenuated IBV replication in host cells. Thus, at least in this case, the deletion was more likely to have been the result of genetic variation due to host adaptation, rather than because this gene mutant already exists in the virus population.

As part of their life cycle, viruses rely on the exploitation of host cell processes and resources for replication. Recent studies of viral life cycles have shown that virus assembly is promoted by tampering with cellular pathways (19–21). In the process of virus replication, viral proteins must interact closely with the host cell mechanism. In addition to playing a role in virus replication or virus assembly, many coronavirus proteins expressed in infected cells are also involved in the interaction between coronavirus and host (22), e.g., by changing the host gene expression or counteracting the host antiviral defense to create an optimal environment for coronavirus replication (23, 24). The interaction between these coronavirus proteins and the host is the key to viral pathogenesis and will ultimately determine the outcome of infection. Therefore, determining whether 5a protein affects the localization and function of host cell proteins is also helpful for understanding how 5a protein performs its function.

First, we studied the cellular sublocalization of 5a protein by using tagged 5a proteins. Under a confocal microscope, EGFP-tagged 5a protein showed obvious cytoplasmic and perinuclear distribution, a pattern which was significantly different from that of control EGFP. Additionally, the localization pattern of Flag-tagged 5a protein was consistent with that of EGFP-tagged 5a protein, indicating that the labeling process did not affect the cellular localization pattern of 5a protein. After the successful construction of Flag-tagged 5a protein, we identified 16 differentially expressed host proteins by co-IP and mass spectrometry, 8 of which are related to the nuclear and cytoplasmic transport and translation of host proteins. 5a has an interesting perinuclear distribution pattern, indicative of a potential role in nucleocytoplasmic protein transport. Protein transport is the basis of cell function and cell survival. In the cellular protein transport system, entry into the nucleus through the nuclear pore complex (NPC) is necessary for many proteins to function. Proteins less than 20 to 40 kDa in size can be passively diffused through the NPC, whereas larger proteins require active promotion by transportins to enter or leave the nucleus. Because nucleocytoplasmic transport plays a key role in the function of many cellular processes, cells have developed elaborate systems to strictly control this process. Unfortunately, errors can occur at every point of the regulatory pathway, and viruses take advantage of this to create an environment conducive to their replication.

The co-IP results proved that HA-labeled TNPO1, TNPO3, XPO1, XPOT, RanBP1, and EIF2B4 proteins could interact with Flag-tagged 5a protein. Transportins, such as TNPO1 and TNPO3, have been shown to have an effect on mitosis and the postmitotic recombination of interphase nuclei, a process that is essential for transcription and replication through cyclins (25, 26). Changes in the function of TNPO1 not only disrupt this process but also affect the genetic characteristics of newly formed cells after mitosis. Nuclear output receptor protein Exportin1 (CRM/XPO1) is the main extranuclear transporter of cargo proteins, recognizing and binding their nuclear export signal (NES), and it acts on mitotic spindles, kinetoplasts (mediating microtubule/kinetoplast interactions necessary for stabilizing chromosome segregation), and centrosomes (preventing chromosome repeats that pose a major threat to genomic stability). The effects of XPO1 on mitosis are mediated by several mitotic targets containing NES sequences. Notably, XPO1 contributes to the localization of mitotic cyclin B, while mitotic cyclin B phosphorylates XPO1 to finely regulate its mitotic function (27). Previous research has shown that XPO1 can mediate the output of viral proteins closely related to respiratory syncytial virus and influenza virus (28, 29). XPO-T and XPO5 participate in eEF1A-mediated nucleoacylation-dependent tRNA and microRNA (miRNA) precursor export pathways and participate in the nuclear and cytoplasmic transport of tRNA. Additionally, XPO-T is involved in the nuclear and cytoplasmic transport of HIVNef/eEF1A/tRNA complexes in macrophages (30). Ran-binding protein 1 (RanBP1) is a key regulator in the Ran-GTPase cycle. RanBP1 is found in all organisms and is highly conserved across different species that contain Ran-binding domains. Furthermore, RanBPl plays an important role in nuclear and cytoplasmic transport, centrosome assembly, microtubule polymerization, spindle assembly, and apoptosis (31–33). Translation initiation factor EIF2B subunit δ is a protein encoded by the human gene EIF2B4. It is a eukaryotic translation initiation factor closely related to cell survival. This gene belongs to the eukaryotic translation initiation factor 2 (EIF2) gene family and is considered a candidate host gene for controlling virus replication. Notably, infection with Newcastle disease virus can lead to the inactivation of EIF2B in chicken tissues (34).

Our results showed that 5a protein could cause changes in the localization of host proteins. Because 5a protein lacks an NES sequence, we speculated that 5a protein might hijack host proteins to create a favorable environment for other IBV proteins with a nuclear localization signal (NLS). Previous studies have shown that there are nuclear localization and nuclear output sequences on the N protein of coronavirus, and the host nuclear shuttle system is used for cytoplasmic and nuclear transport (35–37). Although *Coronaviridae* family N proteins have no discernible homology, they share a nucleolar-cytoplasmic distribution pattern. Despite CoV N protein harboring three putative nuclear NLS motifs (35, 36), this protein is distributed predominantly in the cytoplasm of SARS-CoV-infected and N gene-transfected cells (37), which is consistent with our observations in the present study. However, an identical nucleolar-cytoplasmic localization pattern is observed for the N of coronavirus groups I, II, and III, as exemplified by transmissible gastroenteritis virus (TGEV), mouse hepatitis virus (MHV), and IBV, respectively (38, 39). It has been hypothesized that the caspase activation during the apoptosis of SARS-CoV infection leads to the cleavage of N at particular sites. Some of the cleavage products may contain an active NLS, leading to its nuclear translocation, which may disrupt or usurp nuclear function, whereas the self-association of some N molecules may protect N from cleavage by caspases such that they can remain in the cytoplasm and participate in virus assembly (35). N localization to the nucleus/nucleolus implies that this protein may be involved in regulating the cell cycle, possibly by delaying cell growth to promote optimal intracellular conditions for virus assembly and sequestering ribosomes for the translation of viral proteins or by participating in the modulation of nucleolar functions, as a strategy to optimize virus replication (35). Although coronavirus replication is generally accepted to occur in the cytoplasm of infected cells (40), for IBV an intact cell nucleus has been proposed to be necessary for virus replication (41). However, the possible role of the nucleolus in IBV replication has not been elucidated, and why the N protein would localize to this structure remains unknown. Here, we observed the accumulation of N in the cytoplasm and nucleus; after cells were cotransfected to express 5a or host nucleocytoplasmic transport proteins (TNPO1, TNPO3, XPO1, XPOT, RanBP1, or EIF2B4), high levels of fluorescence intensity were seen in the nucleolus. We speculate that 5a may relocate N into the nucleus via hijacking host proteins, as part of a virus strategy to control both host cell and virus subgenomic RNA (sgRNA) translation by associating with ribosomal subunits and to create a favorable environment for virus replication.

In conclusion, this study found that a 5a gene deletion in the IBV genome affected virus replication and sgmRNA synthesis in the early stage of the virus life cycle, leading to decreases in protein synthesis and in offspring virus assembly and release in CEK cells. We also identified that IBV 5a protein can interact with multiple host nuclear and cytoplasmic transport- and translation-related proteins in host cells, which in turn might interact with IBV N protein and affect its cellular localization. This work provides a comprehensive overview of the role of IBV accessory protein 5a as well as an important theoretical basis for the study of the interactions between coronavirus and host cell proteins.

## MATERIALS AND METHODS

### Viruses and cells.

The IBV strain rYN and 82-nt deletion strain rYN-Δ5a were constructed and rescued using a vaccinia virus-based reverse genetic system in a previous study and preserved at –80°C for further use (10). African green monkey kidney cells (Vero) (CRL-1586; ATCC) and human embryonic kidney (HEK) 293T cells (CRL-11268; ATCC) were preserved in our laboratory. CEK cells were prepared from 18-day-old SPF chicken embryos as previously described (42). SPF embryonated eggs were purchased from Beijing Boehringer Ingelheim Vital Biotechnology Co., Ltd., (Beijing, China). All cells were cultured in Dulbecco’s modified Eagle’s medium (DMEM) (catalog no. 12100-046; Gibco) supplemented with 10% fetal bovine serum and 1% penicillin/streptomycin.

### Early viral replication comparison using a flow cytometry assay.

To investigate the effect of the 5a gene on specific steps of the virus replication cycle, we first tested whether the 5a 82-nt deletion restricts viral entry and early-stage replication, by comparing the percentages of rYN- and rYN-Δ5a-infected CEK cells, as assessed by flow cytometry, at different time points. Confluent CEK cells in six-well plates were inoculated with 200 μL of phosphate-buffered saline (PBS) containing 10^8^ copies of rYN-Δ5a or rYN, and cells from each group (three wells each) were collected separately at 2, 4, 6, 8, 12, 16, 20, and 24 hpi. To digest the cells, 500 μL of trypsin-EDTA was added to each well; this was then neutralized with 1 mL of PBS plus 8.3% fetal bovine serum. The cells were centrifuged for 5 min at 500 × *g*, and the resulting cell pellet was resuspended in 200 μL of PBS and transferred to a 96-well round-bottomed plate. The cells were then fixed overnight on ice with 200 μL of 1× FoxP3 fix/perm buffer (Cytofix/Cytoperm with Golgplug kit, catalog no. 555028; BD Pharmingen, San Diego, CA, USA). After being washed three times with 200 μL of perm/wash buffer, the cells were stained for 30 min on ice in the dark with 100 μL of mouse anti-IBV N monoclonal antibody (MAb) (Hytest, Finland) diluted 1:1,000 in perm/wash. After being washed another three times with 200 μL of perm/wash buffer, the cells were incubated with anti-mouse IgG(H+L) F(ab′)_2_ fragment (Alexa Fluor 488 conjugate) (Cell Signaling Technology, Danvers, MA, USA) for 1 h on ice in the dark. Finally, the cells were resuspended in 200 μL of perm/wash and transferred to flow cytometry tubes for the detection of fluorescein isothiocyanate (FITC)-positive cells on a BD FACSCanto II (BD Biosciences, San Jose, CA, USA).

### Comparison of sgmRNA synthesis and genome replication.

CEK cells were infected with identical doses (200 μL of PBS containing 10^8^ copies) of rYN or rYN-Δ5a. The total RNA of each well was extracted at 1, 12, 16, 20, 24, 36, 48, or 60 hpi and then analyzed by qRT-PCR. The primers for detecting different lengths of sgmRNA were designed based on the sgmRNA production characteristics of coronavirus. The forward primer was designed downstream of the 5′ leader sequence of the IBV genome, while the reverse primers were specific for S, 3ab&E, M, 5ab, and N gene sgmRNA. The primer sequence specific to the 1ab region of the whole genome was designed to test the replication level of the whole genome, and the upstream primer was the same as that for the sgmRNA detection assay. The downstream primers were specific for the nsp2 gene in 1a (Table 2). The copy number of each gene sgmRNA and the genome RNA was calculated based on the standard curve constructed for each gene. All detection assays were repeated three times.

**TABLE 2:** PCR amplification primers used in this study

Primer name	Sequence (5′–3′)
Leader-F	AAATATATATCATACATACTAGCCTTGCGC
1ab-R	GGAACACCTATTGGGACCAGC
S-sgmRNA-R	CTACATAGTGCAAACAAAACAGTCAC
3ab-sgmRNA-R	CGCACTCTCTAAAACAACTTAAAGCA
E-sgmRNA-R	GAAAACTGCCATTCTCTTCTAGCG
M-sgmRNA-R	ATATTCCTTGAAGAGCAGAATAGCCTG
5ab-sgmRNA-R	CGCTTGGTACCGTGCTCTAAA
N-sgmRNA-R	AGTGCCTGAAACCATGATGCA
Flag-5a-F	AAGGACGACGATGACAAGATGAAATGGCTGAATAGTTTAGG
Flag-5a-R	GGCGGCCAAGCTTCTGCAGGTCCTATACCAGCGATTGAGCG
5a-EcoRI-infu-F	GTTCTATCGATTGAATTCATGAAATGGCTGAATAGTTTAGG
pKR5-EGFP-SalI-R	GCCAAGCTTCTGCAGGTCGACCTACTTGTACAGCTCGTCCATGCC

### Western blotting assay for detecting the protein synthesis level of IBV N.

CEK cells were infected with identical doses (200 μL of PBS containing 10^8^ copies) of rYN or rYN-Δ5a. At 12, 24, 36, or 48 hpi, the cells were washed with cold PBS and harvested in 80 μL of 2× lysis buffer. After being incubated on ice for 30 min, the cells were sonicated for 1 min and then boiled. Equal protein amounts were separated by SDS-PAGE, proteins were detected via Western blotting using specific antibodies against IBV N (Hytest, Finland) and β-actin (Merck Millipore, Burlington, MA, USA) and the ChemiDoc imaging system (Bio-Rad, Hercules, CA, USA). Protein signal intensities were normalized and quantified using ImageJ software (NIH, Bethesda, MD, USA).

### Levels of viral offspring released by CEK cells infected with YN or rYN-Δ5a.

CEK cells were infected with identical doses (200 μL of PBS containing 10^8^ copies) of rYN or rYN-Δ5a. At 1, 12, 16, 20, 24, 36, 48, 60, 72, or 84 hpi, the supernatants of each well were collected and used for qRT-PCR analysis and Western blotting. Primers specific for the whole-genome 1ab region were used for qRT-PCR analysis. The genome copy number was detected in three repeated assays. The total protein in the supernatant was extracted by the methanol-chloroform precipitation method, and the protein expression levels of N were detected as previously described.

### Construction of expression plasmids carrying tagged 5a.

IBV rYN viral RNA was extracted by using an RNAprep pure tissue kit (Tiangen Biotech, Beijing, China) in accordance with the manufacturer’s instructions. Reverse transcription was conducted at 37°C for 1 h, with 3 μg of total RNA, 1 μL of random hexamer primers (500 μg/mL; Promega, Madison, WI, USA), and 0.5 μL of M-MLV reverse transcriptase (200 U/μL; Promega). Next, the 5a ORF was amplified by PCR with Premix PrimeSTAR HS DNA polymerase (TaKaRa Biotechnology Co., Ltd., Japan). To create pRK5-5a-EGFP, in which 5a is fused at its C terminus with EGFP, we used a forward primer that contained an EcoRI site and the 5′ end of the 5a gene. The reverse primer contained a BamHI site and the 3′ end of the 5a gene, but no 5a stop codon to allow readthrough transcription (Table 2). The restriction enzyme-digested PCR product was cloned into pRK5-EGFP with a mutated EGFP start codon (TTG instead of ATG). To create pRK5-5a-Flag, a 5a sequence synthesized with a Flag-tag at the C terminus was cloned into the expression plasmid pRK5. The sequences of the cloned expression constructs were confirmed by using Sanger sequencing. At 24 h before transfection, Vero cells were seeded into six-well plates at a density of 10^6^ cells per well and cultured overnight. In accordance with the StarFect high-efficiency transfection reagent (GenStar, Beijing, China) instructions, 2 μg of PRK5-EGFP, PRK5-5a-EGFP, PRK5-Flag, or PRK5-5a-Flag was transfected into the cells. At 48 hpi, the cells were lysed, and the total cellular protein was extracted. The expressions of EGFP-tagged 5a protein and Flag-tagged 5a protein were detected by Western blotting with anti-EGFP mouse MAb (Sangon Biotec, Shanghai, China) and DYKDDDDK Tag mouse MAb (which binds to the same epitope as anti-Flag MAb; Cell Signal Technology, MA, USA), respectively.

### Immunofluorescence analyses.

Vero cells were grown on 12-mm coverslips and then transfected with 1 μg of pRK5-EGFP, pRK5-5a-EGFP, or pRK5-5a-Flag. At 24 h posttransfection, the cells were fixed with 4% paraformaldehyde. Following permeabilization using 0.2% Triton X-100 and subsequent blocking with PBS buffer containing 1% fetal calf serum, 5a protein was detected using anti-EGFP mouse MAb or DYKDDDDK Tag mouse/rabbit MAb, followed by incubation with secondary antibody conjugated to Alexa-488/555 (Life Technologies, Gaithersburg, MD, USA). Fluorescence signals were captured with an A1 confocal microscope (Nikon, Tokyo, Japan).

### Screening for host proteins that interact with 5a.

Co-IP techniques coupled with liquid chromatography tandem mass spectrometry (LC-MS/MS) were used to screen cellular proteins for their ability to interact with IBV 5a protein. Vero cells were seeded in 10-cm cell dishes, and when the cell density reached 80% to 90%, the cells in each dish were transfected with 20 μg of pRK5-5a-Flag or the empty vector pRK5-Flag, which was used as a control. At 36 h posttransfection, cells were collected and lysed. Host cellular proteins that interact with the IBV 5a protein were immunoprecipitated using an anti-Flag M2 affinity agarose gel (Sigma-Aldrich Corp., St. Louis, MO, USA), then identified by LC-MS/MS, and finally analyzed by GO annotation (43). The Gene Multiple Association Network Integration Algorithm (GeneMANIA, http://www.genemania.org/) was used to reveal physical and genetic protein-protein interactions for protein-protein interaction (PPI) network construction and visualization (44).

### Interaction between IBV 5a protein and host proteins.

To confirm the interactions between IBV 5a protein and the identified host proteins, plasmids for HA-tagged versions of the host proteins TNPO1, TNPO3, XPO1, XPOT, RanBP1, and EIF2B4 were constructed with the PRK5 vector and transfected into 293T cells. At 36 h posttransfection, the cells were harvested, and the expression and protein size of the host proteins were verified by Western blotting with anti-HA-Tag (C29F4) rabbit MAb (Cell Signal Technology). Additionally, the HA-tag host protein plasmids were each transfected into Vero cells seeded on 12-mm coverslips. Host proteins were detected using the anti-HA-tag (C29F4) rabbit MAb, followed by incubation with a secondary antibody conjugated to Alexafluor-488 or Alexafluor-555 (Life Technologies). Fluorescence signals were captured with a Nikon A1 confocal microscope (Nikon).

The interactions between IBV 5a protein and the identified host proteins were further confirmed by co-IP assays and confocal microscopy. After verifying the successful expression of HA-tagged TNPO1, TNPO3, XPO1, XPOT, RanBP1, and EIF2B4, the HA-tagged host protein plasmids were cotransfected with PRK5-Flag-5a into 293T cells. At 36 h posttransfection, the cells were collected, lysed, and centrifuged. The target protein in the resulting supernatant was captured by an anti-Flag M2 affinity agarose gel (Sigma-Aldrich), and the expression of host proteins in the immunoprecipitation samples was detected with anti-HA antibody. The localizations of 5a protein and the six host proteins in Vero cells after cotransfection were detected using a Nikon A1 confocal microscope, and the colocalization phenomenon and localization changes were observed.

### Nuclear and cytoplasmic protein extraction.

CEK cells were infected with identical doses (200 μL of PBS containing 10^8^ copies) of rYN or rYN-Δ5a. At 24 h, the cells were washed with cold PBS and transferred to a 1.5-mL microcentrifuge tube by centrifuging at 5,000 × *g* for 5 min. The cells were lysed, and cytoplasmic and nuclear extracts were prepared using a nuclear and cytoplasmic protein extraction kit (Beyotime Biotech, China) according to the manufacturer’s instructions.

### Western blotting assay for detecting the protein synthesis level of IBV N.

CEK cells were infected with identical doses (200 μL of PBS containing 10^8^ copies) of rYN or rYN-Δ5a. At 12, 24, 36, or 48 hpi, the cells were washed with cold PBS and harvested in 80 μL of 2× lysis buffer. After being incubated on ice for 30 min, the cells were sonicated for 1 min and then boiled. Equal protein amounts were separated by SDS-PAGE, and proteins were detected via Western blotting using specific antibodies against IBV N (Hytest, Finland), β-actin (Merck Millipore, Burlington, MA, USA), α-tubulin antibody (Abcam, Cambridge, UK), and histone H3 antibody (Abcam). Images were taken with the ChemiDoc imaging system (Bio-Rad, Hercules, CA, USA). Protein signal intensities were normalized and quantified using ImageJ software (NIH, Bethesda, MD, USA).

### Investigation of proteins affecting the localization in Vero cells.

We further studied the effect of IBV 5a protein on the entry of IBV N into the nucleus. We cloned the full-length N gene of IBV into PCI-neo to construct the eukaryotic expression vector PCI-N. Vero cells were transfected with PCI-N alone or cotransfected with PCI-N and PRK5-Flag-5a. The localization pattern of IBV N was observed by confocal microscopy. We also cotransfected Vero cells with PCI-N and various host protein expression vectors and then observed via confocal microscopy whether there were similar localization changes.

### Statistical analysis.

Statistical significance was evaluated using two-way analysis of variance (ANOVA) tests adjusted for *post hoc* analysis, followed by Bonferroni’s multiple-comparison tests. Individual data points from each independent experiment were used for the calculation of significance. The number of independent experiments is indicated in each figure legend.
